# Dermatoscopy of primary localised cutaneous amyloidosis ‐ A cross‐sectional study in a setting of South Asian public dermatology department

**DOI:** 10.1002/ski2.316

**Published:** 2023-11-27

**Authors:** Prajwal Pudasaini, Sushil Paudel, Sagar Gc, Sadiksha Adhikari, Prashanta Pudasaini, Kinnor Das, Paweł Pietkiewicz

**Affiliations:** ^1^ Department of Dermatology Civil Service Hospital Kathmandu Nepal; ^2^ Department of Dermatology Gandaki Medical College and Teaching Hospital Pokhara Nepal; ^3^ Kathmandu Medical College Sinamangal Kathmandu Nepal; ^4^ Apollo Clinic Silchar Assam India; ^5^ Dermatology Private Practice Poznań Poland

## Abstract

**Background:**

Amyloidosis, deposition of misfolded protein in body, is a fairly common condition. The deposition of misfolded proteins in skin which occurs in absence of systemic comorbidities, namely Primary Cutaneous Amyloidosis (PCA) is also a well‐known entity in skin of colour patients of Asian subcontinent. Primary Cutaneous Amyloidosis is usually diagnosed with good clinical acumen and typical clinical phenotype and involved site. Dermoscope has been used as an adjunct non‐invasive tool to confirm cases with diagnostic uncertainty and in those in whom biopsy is deferred. Typical dermoscopic features of PCA helps differentiate it from other pigmentary dermatoses and avoids unwanted invasive biopsies and investigations especially in resource poor settings with financial constraints.

**Objectives:**

This study aims to identify and corroborate clinically, typical dermoscopic features in PCA in 42 patients which includes Macular Amyloidosis (MA) and Papular Amyloidosis (PA) predominantly in skin of colour patients from government based hospital of a south east Asian country.

**Materials and methods:**

Patients with classic clinical features of PCA were selected. Primary Cutaneous Amyloidosis was subclassified into MA or PA and their corresponding clinically corroborative dermoscopic features were enlisted respectively. All patients (treatment naïve and previously treated), who consented to participate in the study were included. Patients were diagnosed based on the prototypical clinical features. Dermoscopy was done using DermLite III DL3N Polarised and Fluid Dermoscope w/PigmentBoost Brand (3Gen, DermLite LLC, San Juan Capistrano, CA, USA) and images were obtained to create digital dermoscopy system by attaching camera‐equipped mobile device via an optional connection kit (Redmi Note 11, MIUI version 13.0.5, CHINA) and the findings were enlisted concurrently.

**Results:**

In this study of dermoscopic findings of PCA, 42 patients were evaluated for their clinical lesions along with its corroboration with the dermoscopic features. Macular Amyloidosis was seen in 30 patients and 12 patients had typical cutaneous phenotypic and dermoscopic feature of PA. The most common dermoscopic finding seen in patients with MA was shiny to dull white, circular or oval central hub surrounded with halo of light brown dots. Most common configuration of brownish pigmentation around central hub was fine streak type. Also eccrine clues were seen in some cases of MA, which was a unique finding. Similarly in the PA subtype, the central hub was replaced by scar like structureless translucent white area surrounded by brownish black dot like structures, especially in those with large and thick plaques.

**Conclusion:**

Dermoscopic findings of PCA and their clinical corroboration is a much‐needed aspect in treating patients with pigmentary disorders and in those with skin of colour, especially in developing countries. Utilization of dermoscope in clinical settings of low income countries and in government based hospitals will decrease the add on economic burden of invasive diagnostic modalities like biopsy and other inadvertent tests done to rule out pigmentary conditions.

## Graphical abstract

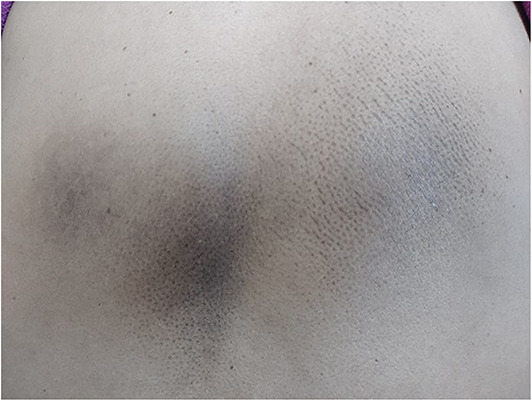

1

**What is already known about this topic?**

- Dermoscopic features of primary localised cutaneous amyloidosis in global context

**What does this study add?**

- This is the first study from Nepal, evaluating dermoscopic features of cutaneous amyloidosis in skin of colour patients.
- Utilization of dermoscopy in government based hospital in resource poor setting

## 2 INTRODUCTION

Amloidosis is a heterogeneous cluster of acquired or hereditary diseases characterised by deposition of amorphous misfolded proteins in body tissues and organs including heart, kidneys, digestive system, nervous system and the skin, impairing and disrupting their functions. The disorder can be further categorised into particular subtypes based on the misfolded protein type and organ involvement. Contrary to systemic amyloidosis where proteinaceous material forming undegradable β‐sheeth fibrils originates from circulating paraproteins or immunoglobulin chains, amyloid aggregates in primary localised cutaneous amyloidosis (PCLA; exclusively affecting the skin) derive from apoptotic keratinocytes, hypothetically due to intense scratching.^1^, ^2^, ^3^, ^4^ The disease is fairly common in Asians and Latin Americans, yet the data on the prevalence and clinical patterns in Caucasian population is scant.^5^, ^6^, ^7^, ^8^

There are 4 recognized primary localized cutaneous amyloidosis (PLCA) variants: lichen amyloidosis (LA; or Papular Amyloidosis (PA)), macular amyloidosis (MA), nodular amyloidosis, biphasic amyloidosis (BA; LA/MA overlap), with the first two being increasingly more common.^7^, ^9^ LA clinically presents as grouped, localised or disseminated, discrete, skin‐coloured to brownish‐black keratotic papules, usually affecting the frontal aspect of lower legs, whereas MA manifests as poorly‐defined, rippled or reticulated brownish‐to‐black macule predominantly affecting the upper back and less frequently lower legs.^7^, ^10^ Of note, chronic localised pruritus in MA is followed by frictional hyperpigmentation, which may be the cause of psychological distress, especially in young females in a skin of colour. Hence, timely identification and management of PLCA may lead to improved Health Related Quality of Life of patients.^11^ Dermatoscopy might be a valuable aid in resource‐poor settings if cases where PLCA is considered as a clinical differential diagnosis, especially in skin of colour.^6^ The disease shares morphological similarities with other pigmentary conditions such as Lichen Simplex Chronicus, Prurigo Nodularis and Post Inflammatory Hyperpigmentation. Use of the dermoscope will defer use of invasive diagnostic modalities such as biopsy, which is the common and important diagnostic procedure done in dermatology clinics.^12^, ^13^, ^14^, ^15^ Avoidance of diagnostic procedures such as‐biopsy will tend to decrease the complications associated with this invasive procedure and decrease the economic burden of unwanted diagnostic tests in patients with financial constraints in resource poor settings.^16^, ^17^

Because of financial constraints, unavailability of modern dermoscope and lack of training on dermoscopy, its utilization has not been as rampant as it should be in resource‐poor settings like ours. This is an initial attempt by practicing clinicians of government based hospital of Nepal in evaluating dermoscopic features of Primary Cutaneous Amyloidosis (PCA) and its clinical corroboration.

## 3 MATERIALS AND METHODS

We conducted a cross‐sectional study after approval from Institutional Review Board (IRB) to assess dermoscopic features of PCA in 42 patients who visited out patient department of Civil Service Hospital from January 2022 to December 2022. Fourty two patients of PCA, thirty of MA subtype and twelve of PA subtype were included in this study. All patients (treatment naïve and previously treated), who consented to participate in the study were included. All the patients were diagnosed based on the prototypical clinical features. Dermoscopy was done using DermLite III DL3N Polarised and Fluid Dermoscope w/PigmentBoost Brand (3Gen, DermLite LLC, San Juan Capistrano, CA, USA)and images were obtained to create digital dermoscopy system by attaching camera‐equipped mobile device via an optional connection kit (Redmi Note 11, MIUI version 13.0.5, CHINA). Typical dermoscopic patterns including central hub, radiating spikes and colour of the hub was noted and corroborated with the MA and PA subtypes respectively. Seventy percent Ethanol was used as contact fluid to increase penetration for visibility of epidermal and dermo‐epidermal structures. The contact polarised mode of the dermoscope was used for observation.

## 4 RESULTS

In this study of 42 patients of PCA, 30 patients of MA and 12 patients of PA were evaluated.

Among the 42 patients enroled in the study, 28 were females and 14 were males with a female to male ratio of 2:1. Mean age of the patient was 38 years and the age range was in between 26 and 62 years. The duration of lesions ranged from 5 months to 9 years with the mean duration of 2.5 years. More than half of the patients (56.5%), experienced pruritus over the lesion site. Eighteen patients (42.85%) had involvement of more than two sites including back, upper arms, forearm, elbow, shin and chest. Back and lower legs were the commonest sites involved in 67% of the patients.

Patients were diagnosed with typical clinical phenotypic features and diagnoses were corroborated with dermoscopic structures such as central hub, spokes, configuration and these patterns were corroborated with respective clinical subtypes.

## 5 MACULAR AMYLOIDOSIS

**FIGURE 1:**
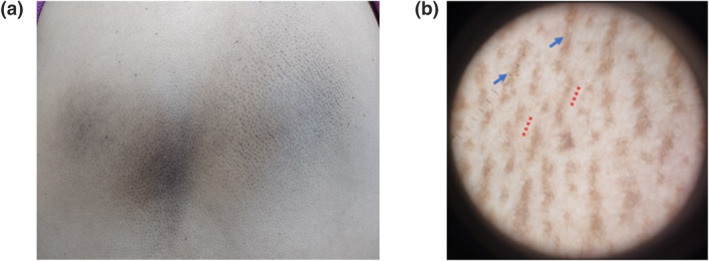
Case of Macular Amyloidosis (MA) (a) Clinical image: brownish black rippled macule over upper back in the mid, inter scapular and infra scapular region (b) Dermoscopic image: dull white, circular central hub *(red dotted lines)* with halo of light brown dots along with fine streak like configuration of brownish pigmentation *(blue arrow)* around central hub.

**FIGURE 2:**
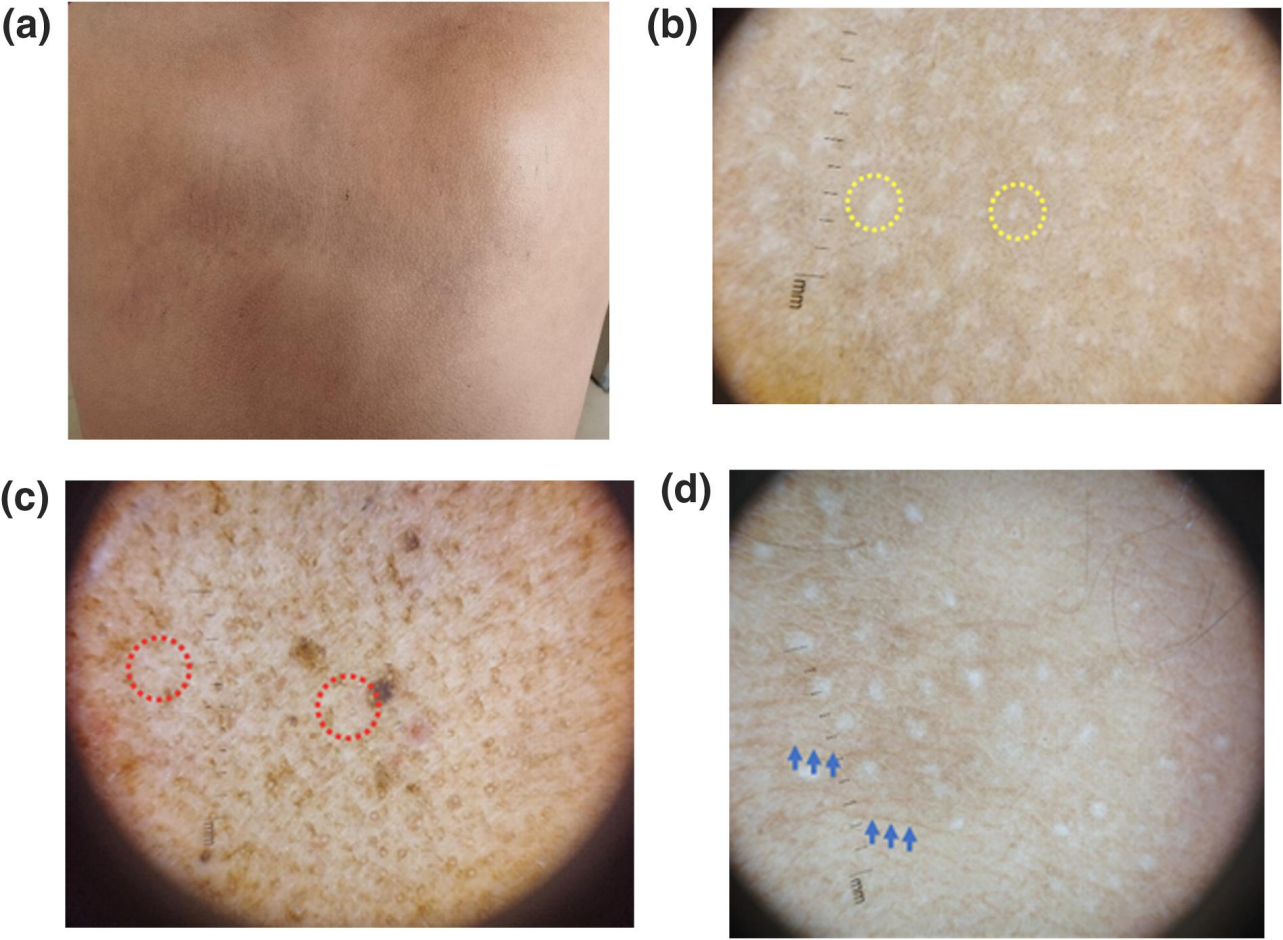
Macular Amyloidosis (MA) (a) brownish black macule over upper back. (b), (c) Dermoscopic feature of whitish central hub surrounded by brownish halo along with leaf like configuration *(yellow dotted circle in image b and red dotted circle in image c)* (d) fine streak like configuration of the halo around the central hub *(tip of blue arrows)*.

In all patients with this subtype of amyloidosis, upper back was involved congruously. Dermoscopic pattern showed shiny to dull white, circular to oval central hub with halo of light brown dots in 28 patients (Table 1). Configuration of brownish pigmentation around central hub showed fine streak in 24 patients, bulbous pattern in 4 patients and leaf like in 2 patients. Also eccrine clues were seen in cases of MA. Figures 1 and 2 depict clinical and dermoscopic images of MA. Furthermore, closest mimics of PLCA with their corresponding dermoscopic and histopathological evaluation findings have been elucidated (Table 2).

**TABLE 1:** Clinical types of Primary Cutaneous Amyloidosis (PCA) (Macular and Papular) and their corresponding dermoscopic features (*n* = number of patients).

Primary cutaneous amyloidosis (PCA) types	Site	Dermoscopic pattern	Structure of central hub	Configuration of brownish pigmentation around central hub
**Macular amyloidosis (MA)** (*n* = 30)	Upper back (*n* = 30)	Central hub (*n* = 20)	Shiny to dull white, circular or oval with halo of light brown dots (*n* = 28)	Fine streak (*n* = 24)
		Linear scar like whitish structureless area (*n* = 10)	Brownish black(*n* = 2)	Leaf like (*n* = 2)
		Perifollicular reduced pigmentation with light coloured halo (*n* = 2)		Bulbous (*n* = 4)
**Papular amyloidosis (PA)** or lichenoid (*n* = 12)	Lower leg (*n* = 10)	Central hub replaced by scar like structureless translucent white area surrounded by brownish black dot like structures, in those with large and thick plaques (*n* = 11)	Brownish black = 1	
	Back (*n* = 2)	Perifollicular reduced pigmentation with light coloured halo (*n* = 2)		

**TABLE 2:** Table depicting differential diagnoses of the Primary Cutaneous Amyloidosis (PCA) along with their corresponding dermoscopic and histopathological features (*n* = number of patients).

	Clinical features^18^	Dermoscopy^19^, ^20^, ^21^	HPE^22^, ^23^
Post inflammatory hyperpigmentation	After the initial lesions healed, hyperpigmented patches appear at the affected area. The colouration of the lesions might vary from a light brown to a dark black.	Homogeneous pigmentation, bluish‐grey areas, sharp margins	Increased melanin, epidermal hyperplasia, melanin‐laden macrophages, dermal inflammatory infiltrate, disrupted melanin distribution
Drug induced hyperpigmentation	Depending upon offending drug	Depending upon offending drug	Increased melanin, epidermal hyperplasia, melanophages in the dermis, altered melanocyte activity, dermal inflammation, basal layer pigmentation, specific drug‐related features
Chromic pityriasis versicolour	Characterised by hyperpigmented oval with fine, scaling that often coalesce into larger patches. The most prevalent sites for PV are seen on the trunk and the upper proximal extremities.	Irregular pigmentation, inconspicuous ridges and furrows	Hyphae and yeast forms, mild inflammation, spaghetti and meatballs appearance, disrupted stratum corneum, follicular involvement, minimal to No suprabasal acantholysis, melanin accumulation
Lichen planus pigmentosus	Macules and patches ranging from brown to grey and oval in morphology. Usually located on the face, the neck, and/or the intertriginous regions	Linear blue or blue‐grey dots as annular granular structures, dots, globules, and blotches (black/brown/violet) as predominant pigment structures	Increased basal layer pigmentation, vacuolar degeneration, band‐like infiltrate, melanin incontinence, lichenoid interface dermatitis, spongiosis, perivascular inflammation
Erythema dyschromicum perstans	Macules and patches ranging from brown to grey colour. Located along the lines of the skin cleavage in the trunk	Bluish‐grey globules (red arrows) in a bizarre and irregular arrangement referred as “speckled” appearance.	Spongiosis and hyperkeratosis of epidermis. Basal layer hyperpigmentation.
		EDP can be differentiated from LPP on dermoscopy by its predominant blue/grey colour and more homogeneous dot size and arrangement.	Lymphocytic infiltrate. Lichenoid interface dermatitis. Melanophages in dermis. Occasional eosinophilic infiltrate. Absence of vasculitis.
Poikiloderma of civatte	Atrophic areas that are reddish‐brown in colour usually over photo exposed areas	Vascularity, follicular plugging, and reticulated or structureless hyperpigmentation are appreciated. Vessels may appear as dots and globules or irregular lines, uniquely referred to as “spaghetti and meatballs” by errichetti and Stinco.pi	Basal layer hyperpigmentation, dilation of blood vessels, degradation of elastin, lichenoid interface dermatitis, melanophages and absence of epidermal atrophy
Schamberg disease	Usually present over the lower limbs. Irregular maculo popular lesions of orange‐brown colour. The lesions are chronic and persist for years	Red globules and red dots, coppery brown background, brown lines reticular and subtle brown dots	Minimal epidermal changes, dilatation of upper dermal capillaries, pervascular inflammation, hemosiderin‐laden macrophages and absence of vasculitis
Pigmented contact dermatitis	Pruritic erythematous macules and patches, with overlying scaling. Seen over place of contact with allergen	Pseudonetwork, blue/grey dots, and telangiectasias	Increase in the number and the activation of melanocytes in the basal layer, in addition to typical interface changes associated with dermal pigment incontinence, a superficial lymphocytic infiltrate, and vascular proliferation
Exogenous ochronosis	Brown macules and patches ranging from light to dark in colour, with caviar‐like papules (colloid milium) and atrophic, hypopigmented macules scattered throughout.	Globular, curvilinear, or “worm‐like” structures. Telangiectasias obliteration of follicular openings scattered hypopigmented areas	Pigment incontinence, solar elastosis, ochre pigment, ‘banana‐shaped’ fibres in papillary dermis, and eventually degeneration of the collagen; colloid milium and/or granulomas

Abbreviations: EDP, erythema dyscrhomicum perstans; HPE, histopathological evaluation; LPP, lichen planus pigmentosus; PV, pityriasis versicolour.

## 6 PAPULAR OR LICHEN AMYLOIDOSIS (PA)

**FIGURE 3:**
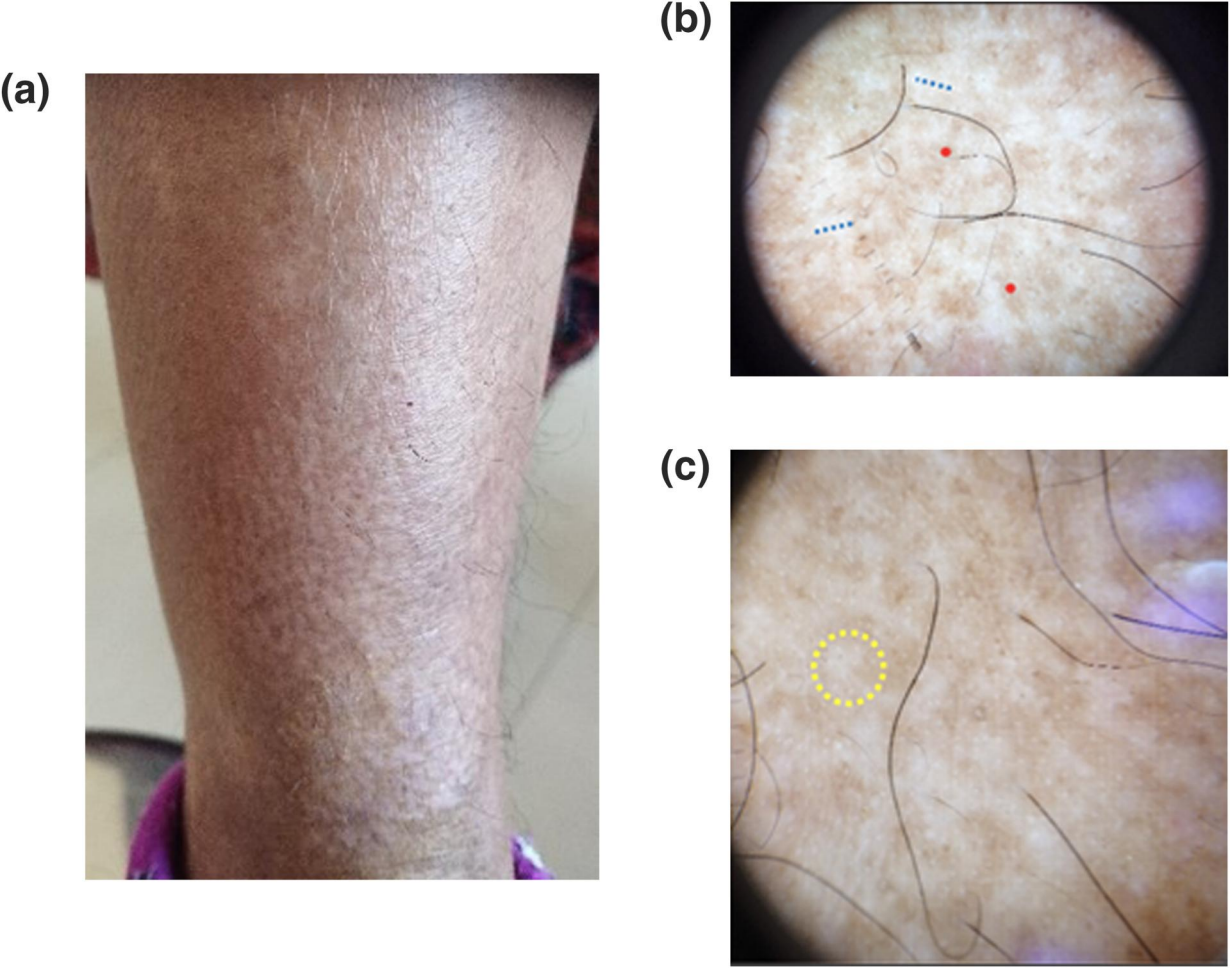
Cases of Papular Amyloidosis (PA): (a) brownish black thick keratotic papule in rippling pattern over extensor aspect of lower leg (b) Central hub replaced by scar like structureless translucent white area *(blue dotted area)* surrounded by brownish black dot *(red dot)* (c) perifollicular reduced pigmentation with light coloured halo *(yellow dotted circle).*

**FIGURE 4:**
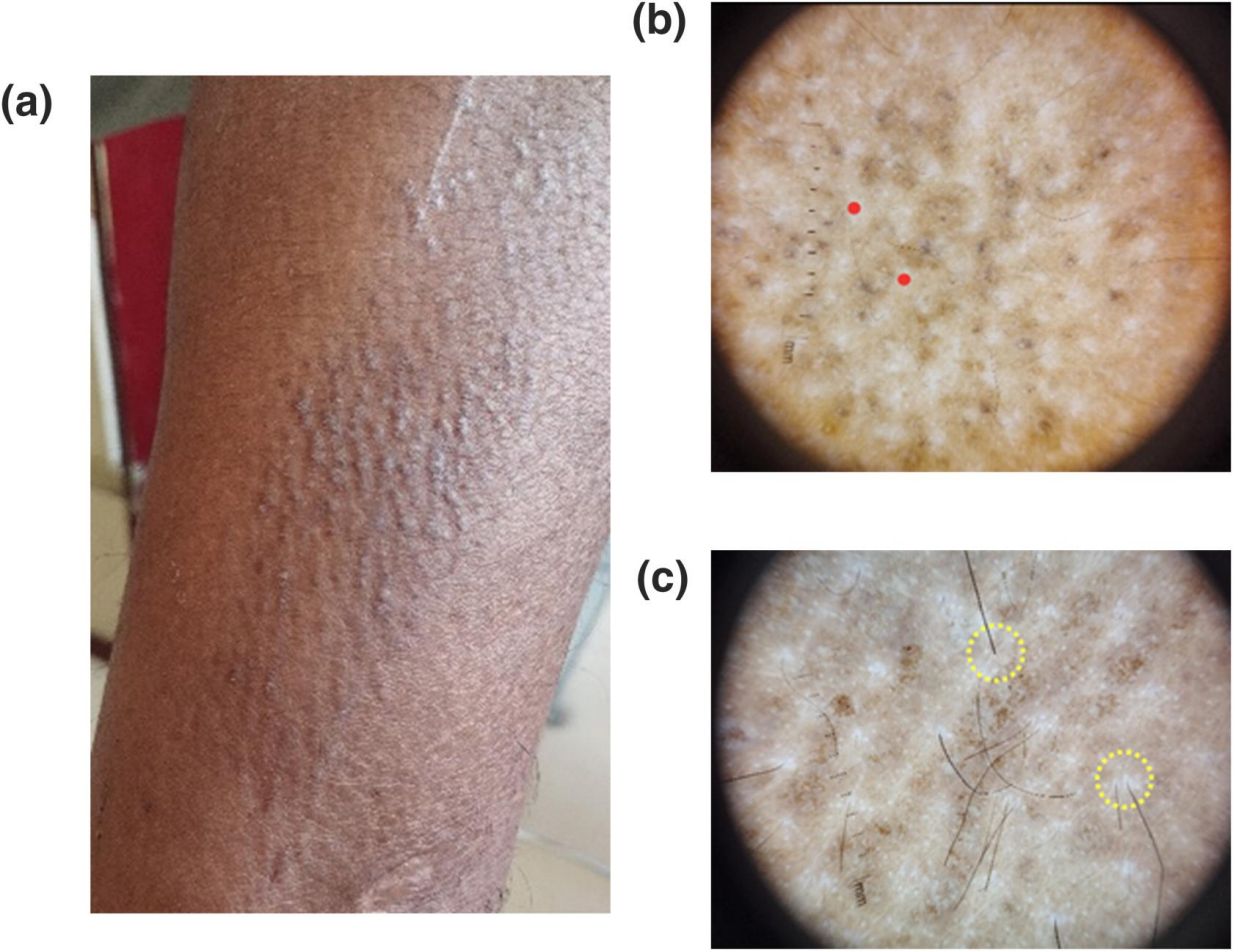
Case of Papular Amyloidosis (PA) (a) brownish black thick keratotic papule with scale in rippling pattern over lateral aspect of lower leg (b) Central hub interspersed along scar like structureless translucent white structure *(red dots)* (c) perifollicular reduced pigmentation with light coloured halo (*yellow dotted circle*).

Among 12 patients, 10 patients with PA had involvement of lower leg and back was involved in two of them. In those with thick lichenoid papule‐central hub was replaced by scar like structureless translucent white area surrounded by brownish black halo of dot like structures (Table 1) (Figure 3). Perifollicular reduced pigmentation with light coloured halo was seen in two patients (Figures 3 and 4).

## 7 DISCUSSION

Utilization of modern diagnostic modalities such as dermoscope in patient treatment is the need of the hour, especially in resource poor setting and government‐based hospital with financial constraints where invasive costly procedure is better deferred. This study corroborates dermoscopic feature of PCA subtype with corresponding clinical features. In this study of dermoscopic findings of PCA of 42 patients, MA was seen in 30 patients and 12 patients had typical cutaneous phenotypic and dermoscopic feature of PA. Similar other studies showed PA as the commonest subtype among the patients reported.^2^(8) Upper back was involved in 28 patients with MA which was consistent to occurrence of MA in upper back in similar other study.^9^ Among patients of PA, lower leg was involved in majority of patients.^10^ The most common dermoscopic feature seen in patients with MA was shiny to dull white, circular or oval central hub with halo of light brown dots around the central hub. Configuration of brownish pigmentation around central hub was fine streak like configuration in 24 patients, followed by bulbous in four patients and leaf like or venation pattern in two. These findings were consistent to dermoscopic features of MA from other study in which the amyloid macule was composed of central hub surrounded by brownish pigmentation and fine streak like configuration around the central hub. Occasionally leaf like configuration of brownish pigmentation could be seen around central hub in some patients.^8^(3) This leaf like or venation pattern, though uncommon can have brownish black pigmented shade over the structure with a background of faint brown tinted area with faded lines.^11^ This recurring pattern of central hub surrounded by radiating pigmented halo is designated as the classical “hub and spoke pattern” seen in cases of MA.^2^ Occasionally the colour of central hub can be brownish black in colour and central hub can be of irregular shape.^12^

In 10 patients with MA, central hub was replaced by linear scar like whitish structureless area, which are seen more often than not, in PA subtypes.^8^ however, this occurrence of central hub is not exclusive to MA subtype and can also occur in LA subtype.^3^ As with central hub pattern which was common to both the subtypes of PCA, perifollicular reduced pigmentation with light coloured halo was also common to both MA and PA subtypes in our study. However, studies have demonstrated the occurrence of perifollicular reduced pigmentation in macular variants only.^11^ In patients with LA, central hub was replaced by translucent white scar like structureless area surrounded by brownish black dot like structures, especially in those with large and thick plaques. Also, perifollicular reduced pigmentation with light coloured halo was seen in a few patients. These findings were consistent with the findings from other study in which dermoscopy of LA showed translucent white scar like centre encircled by irregular brownish black hyperpigmented dots.^3^ Typical lesion of LA in dermoscope shows a double zone pattern characterised by middle whitish‐grey structureless area surrounded by a halo of ridge and fissure area of brownish black pigmentation.^2^ This central whitish structureless area can be of varying morphology, in which case it can either be completely translucent white without demonstrating any dermoscopic patterns/features, or it can resemble a volcaniform crater where there's a halo of brownish dot like structures around the whitish centre. These findings have been corroborated with histopathological diagnoses to further support these diagnostic notions.^3^

## 8 CONCLUSION

Dermoscopic findings of PCA and their clinical corroboration is a much‐needed aspect in treating patients with pigmentary disorders and in those with skin of colour, especially in developing countries. Utilization of dermoscope in clinical settings of low income countries and in government based hospitals will decrease the add on economic burden of invasive diagnostic modalities like biopsy and other inadvertent tests done to rule out pigmentary conditions.

## 9 LIMITATIONS

Because of financial constraints, unavailability of modern dermoscope and lack of training in dermoscopy, its utilization has not been as rampant as it should be in resource‐poor settings like ours.

## AUTHOR CONTRIBUTIONS

**Prajwal Pudasaini:** Conceptualization (lead); Data curation (lead); Methodology (equal); Resources (equal); Software (equal); Validation (equal); Writing – original draft (equal); Writing – review & editing (equal). **Sushil Paudel:** Formal analysis (equal); Methodology (equal); Project administration (equal); Writing – original draft (equal); Writing – review & editing (equal). **Sagar Service Hospital of gc:** Conceptualization (equal); Data curation (equal); Software (equal); Visualization (equal); Writing – review & editing (equal). **Sadiksha Adhikari:** Conceptualization (equal); Data curation (equal); Supervision (equal); Validation (equal); Writing – original draft (equal). **Prashanta Pudasaini:** Data curation (equal); Formal analysis (equal); Validation (equal); Visualization (equal). **Kinnor Das**: Data curation (equal); Formal analysis (equal); Validation (equal); Visualization (equal). **Pawel Pietkiewicz:** Conceptualization (equal); Data curation (equal); Writing – original draft (equal).

## CONFLICT OF INTEREST STATEMENT

None to declare.

## ETHICS STATEMENT

This study has been approved by the IRB of Civil Service Hospital of Government of Nepal, and written informed consent has been taken from all the patients who participated in this study.

## Data Availability

The data that support the findings of this study are openly available in SHD journal with relevant indexing at [https://doi.org/10.1002/ski2.316].
